# Magnetic resonance imaging differentiating benign from malignant bone and soft tissue tumors and assessing Ki-67 expression using APT and DWI tools

**DOI:** 10.3389/fonc.2026.1753717

**Published:** 2026-02-02

**Authors:** Xinxin Liu, Pengxiang Li, Pan Shang, Jing Lu, Kai Ai, Xiaowen Ma

**Affiliations:** 1 MRI Department, Honghui Hospital, Xi’an Jiaotong University, Xi’an, China; 2 Department of Clinical and Technical Support, Philips Healthcare, Xi’an, China

**Keywords:** amide proton transfer imaging, bone and soft tissue tumors, DWI, Ki-67, MRI

## Abstract

**Background:**

Conventional MRI mainly provides the morphologic information of tumors with low specificity, but its ability to distinguish between benign and malignant bone lesions is limited. Amide proton transfer (APT) is a novel MRI tool for detecting amide protons in free proteins and peptides. A few studies have reported on APT in tumors; however, studies on qualitative diagnosis of bone and soft tissue tumors are lacking.

**Purpose:**

This study aimed to investigate the use of APT and diffusion-weighted imaging (DWI) in distinguishing benign from malignant bone and soft tissue tumors. We evaluated the efficiency of APT and DWI sequences in diagnosing bone and soft tissue tumors. In pathology, the Ki-67 index expression reflects the tumor aggressiveness. Malignant tumors are often characterized by increased cell proliferation and increased Ki-67 expression. We assessed the correlation among APT, DWI, and Ki-67 expression.

**Methods and materials:**

The study enrolled 49 patients from June 2023 to January 2024. There were 20 patients in benign tumors, and 29 patients in malignant tumors. The site of the tumors were in the upper and lower extremities. The patients underwent MR scanning, and the diagnosis of bone or soft tissue tumors was confirmed by pathology. The APT, apparent diffusion coefficient (ADC), and exponential apparent diffusion coefficient (EADC) values were measured in the tumor areas. We used APT and DWI to distinguish benign from malignant bone and soft tissue tumors, and analyzed the correlation among APT, ADC, EADC, and Ki-67 expression. We compared the statistical differences in APT, ADC, and EADC values between benign and malignant tumors. The diagnostic efficiencies of APT, ADC, and EADC values and Ki-67 expression were evaluated using the receiver operating characteristic (ROC) curve. Meanwhile, Delong tests were conducted to evaluate the difference in the area under the ROC curve (AUC) among various parameters. The region of interest was outlined manually by 2 senior physicians. The intraclass correlation coefficient (ICC) was used to obtain the interobserver reprehensibility for measuring the APT value.

**Results:**

The ICC was 0.848 in the interobserver agreement, and 95% confidence interval (CI) was 0.607-0.946. The APT value was 2.26% ± 1.07% in benign tumors, and 4.62% ± 1.43% in malignant tumors. The ADC value was 1.33% ± 0.4 ×10_–3_ mm_2_/s in benign tumors, and 1.01% ± 0.55 ×10_–3_ mm_2_/s in malignant tumors. The EADC value was 0.29 ± 0.13 in benign tumors, and 0.40 ± 0.17 in malignant tumors. Statistical differences were observed between benign and malignant tumors in APT (*P* <.001), ADC (*P* = .0032), and EADC (*P* = .019) values. In addition, APT exhibited a positive correlation with Ki-67 [Spearman correlation coefficient: *r* = 0.546, 95% confidence interval (CI): 0.217-0.762, *P* = .002]. In the ROC curve analysis for differentiating benign from malignant tumors, the sensitivity and specificity were respectively 72.41% and 95%, with an APT value of 3.6%;79.31% and 85%, with an ADC value of 1.047×10–^3^ mm^2/^s; and 72.41% and 85%, with an EADC value of 0.34. Combining APT, ADC, and EADC, the sensitivity and specificity were 72.41% and 95%, respectively. The sensitivity and specificity were respectively 100% and 57.14% (*P* <.0001), with an AUC value of 0.794, for APT in assessing the Ki-67 expression; 70.59% and 85.71%, with an AUC value of 0.576, for ADC in assessing Ki-67 expression (*P* <.0001); and 70.59% and 85.71%, with an AUC value of 0.578 for EADC in assessing Ki-67 expression (*P* <.0001).

**Conclusions:**

The MR APT sequence has high qualitative diagnostic efficiency in differentiating benign and malignant bone and soft tissue tumors (when the cutoff value is 3.6%, the sensitivity is 72.41% and the specificity is 95%); the combination of APT, ADC and EADC can improve the diagnostics specificity; the APT value is positively correlated with Ki-67 expression (r = 0.546, P = .002), and can be used as a non-invasive imaging marker to evaluate the proliferative activity of bone and soft tissue tumors. These results need to be further verified by large-sample and multi-center studies.

## Introduction

Bone and soft tissue tumors are pathologically characterized by abnormal cell proliferation and an increased rate and concentration of intracellular protein synthesis (1). Conventional MR T1-weighted imaging (T1WI), T2WI, and fat suppression (FS) T2WI aid in tumor detection and common appearances, including determining the size and shape of the tumor, whether or not with cystic necrosis and bleeding. The contrast-enhanced (CE) MRI is a valuable tool for determining the nature and grade of tumors. However, CE MRI is an invasive method of examination. Patients with allergies to contrast agents or having other contraindications cannot undergo enhanced scanning. Researchers have estimated that 37% of nonenhancing malignant glioma tumors are identified as high grade in the histopathologic assessment (2). A few malignant bone and soft tissue tumors, such as fibrosarcoma and low-grade liposarcoma, demonstrate mild enhancement. Certain benign tumors also manifest obvious reinforcement, such as hemangioma and aneurysmal bone cysts. Therefore, the identification of benign or malignant tumors by CE MRI has certain limitations. CE MRI is a valuable tool for evaluating treatment response. In CE MRI, the reinforcement occurs not only in aggressive tumor tissues but also in treatment-related inflammation, such as postsurgical changes, ischemia, acute radiation effects, and radiation necrosis (3–5). Therefore, distinguishing between tumor recurrence and a positive response to treatment is sometimes difficult.

Functional MRI provides more information on tumor biology. Diffusion-weighted imaging (DWI) reflects water diffusion characteristics, revealing multiple factors such as cell density, vascularity, viscosity of extracellular fluid, and cell membrane integrity (6). Apparent diffusion coefficient (ADC), derived from DWI MRI, is a viable imaging biomarker that can diagnose tumor aggressiveness (7–9).

Amide proton transfer (APT) imaging is a new type of chemical exchange saturation transfer MRI technology. It can detect endogenous mobile proteins and peptides, which resonate at 3.5 ppm^2^. It uses the exchange rate of amide protons in tissue endogenous proteins or polypeptides with water protons to reflect intracellular protein concentration and pH value (10). Compared with traditional perfusion weighted imaging (PWI) and PET-CT, it can conduct non-invasive and quantitative assessment of endogenous mobile proteins and peptides in living tissues without the use of contrast agents, thereby indirectly reflecting the metabolic status of the tissues. Recently, a few researchers have qualitatively diagnosed brain tumors, squamous cell carcinoma of the cervix (11), thoracic lesions (12), prostate tumors (13), and head and neck tumors using APT technology (14). This technology can also predict the pathologic grade of tumors, such as brain tumors (15, 16), rectal cancer (17), and endometrial cancer (18).

Several studies have combined APT and DWI techniques to diagnose tumors. Xu et al. evaluated the preoperative pathologic grade of bladder cancer (19). They concluded that APT and ADC can be used as indicators to predict the histologic grade of bladder cancer, considerably improving diagnostic efficiency. In pathology, the Ki-67 index expression reflects the tumor aggressiveness (20). Malignant tumors are often characterized by increased cell proliferation, large nuclei, abundant macromolecular proteins, and increased Ki-67 expression. Several studies have compared the correlation among APT, DWI, and Ki-67 expression. Li et al. used APT and DWI for assessing p53 and Ki-67 expression in rectal adenocarcinoma (21). However, the relationship between APT and Ki-67 expression in bone and soft tissue tumors has not been reported.

Therefore, in this study, we investigated the diagnostic efficiency of APT, ADC, and EADC in differentiating between benign and malignant bone and soft tissue tumors, and analyzed the correlation of APT, ADC, and EADC values with Ki-67 expression.

We assumed that benign and malignant bone and soft tissue tumors exhibit distinct APT values, with malignant tumors having higher APT values than benign tumors. Additionally, we proposed that combining APT and DWI may enhance diagnostic efficiency and that the APT value correlates with Ki-67 expression.

## Materials and methods

### Study population

This prospective study was approved by the institutional review board. 62 patients, who were clinically suspected of having bone or soft tissue tumors in the upper and lower limbs, were collected from June 2023 to January 2024. The written informed consent was obtained from each patient before the study. The inclusion criteria were as follows: (1) suspected bone or soft tissue tumors on MRI; (2) MRI scanning performed within 2 weeks before surgery; (3) suspected tumors proven to be bone or soft tissue tumors by pathology; (4) no biopsy, invasive treatment, chemotherapy, or radiotherapy before MRI; and (5) without contraindications for MRI scan.

Fatty tissue and cysts and hemorrhage can bring undesirable artifacts (usually hyperintensity) on the APT images. Therefore, when reviewing APT images, we referred to routine T1WI, T2WI and FS images to identify the tumors whether lipoma or cyst or hematoma. The exclusion patients were as follows: (1) bone and soft tissue cyst (*n* = 5); (2) intraosseous ganglion cyst (*n* = 1); (3) bone and soft tissue lipoma (*n* = 5); and (4) soft tissue hematoma (*n* = 2). The total number of patients was 13 included to be excluded. Finally, 49 patients were enrolled in this study. **Table 1** presents the demographic information of the patients.

**Table 1:** Demographic features of the patient population and pathologic diagnosis of tumors.

Data	Benign tumor (*n* = 20)	Malignant tumor (*n* = 29)	*P* value
Age (year)	31.40 ± 17.91	41.76 ± 22.57	.093
Sex (female:male)	7:13	11:18	.834
Bone tumors (*n* = 39)	Nonossifying fibroma (3) Fibrous dysplasia (6) Osteoid osteoma (1) Osteofibrous dysplasia (3) Enchondroma (1) Bone infarction (1)	Chondrosarcoma (1) Osteosarcoma (10) Undifferentiated pleomorphic sarcoma (3) Ewing sarcoma (3) Metastatic tumors (6) Rosai–Dorfman disease (1)	
Soft tissue tumors (*n* = 10)	Schwannoma (3) Giant cell tumor of tendon sheath (1) Hemangioma (1)	Liposarcoma (3) Synovial sarcoma (2)	

### MRI examination

The MRI scan was conducted on 3.0T Ingenia CX System (Philips Healthcare, The Netherlands), with a 16-channel body coil. The MRI scan parameters are provided in **Table 2**. The patients were scanned supine, with both upper limbs placed in front of the chest and lower limbs as close together as possible. Sandbags were placed around the area of interest to help improve the B0 field homogeneity. MRI scans included coronal fat saturation (FS) T2-weighted imaging (T2WI), transverse T1WI and T2WI, and transverse APT and DWI.

**Table 2:** Scan sequences and parameters.

Sequence	TR (ms)	TE (ms)	Field of view (mm)	Matrix	Flip angle	Slice thickness (mm)	Slice gap (mm)	Nex	ETL
T1WI	450	10	220 × 220	276 × 259	90	7	0	1	5
T2WI	2500	100	220× 220	276 × 240	90	7	0	2	18
APT	Shortest	Shortest	130 × 130	268 × 213	90	7	0	2	174
DWI	2521	76	220 × 220	80 × 81	90	7	0	3	N/A

APT, Amide proton transfer; DWI, diffusion-weighted imaging; ETL, echo train length; T1WI, T1-weighted imaging; T2WI, T2-weighted imaging; TE, echo time; TR, repetition time.

### Imaging analysis

The data set was evaluated using a Philips postprocessing workstation (Intellispace Portal; Philips Healthcare). The acquired APT raw data was imported into the IDL (Interactive Data Language) application (Research Systems, CO, USA) for analysis. The Z-spectrum was calculated as a function of the saturation frequency offset. To reduce the inhomogeneity of the B0 field, which were from the conventional magnetization transfer effect and direct saturation of bulk water, we use a B0-corrected Z-spectrum to analyze the magnetization transfer ratio asymmetry (MTRasym). According to the APT technology principle and algorithm, the asymmetry analysis at 3.5 ppm downfield from the water signal was calculated as MTRasym (3.5 ppm) of APT imaging. APT SI % is referred to as the MTRasym value at +3.5 ppm displayed as a percent level (relative to S0) in the final APTw image.

MTRasym (3.5 ppm) = [Ssat (−3.5 ppm) − Ssat (+3.5 ppm)]/S0, which reduce asymmetry analysis of magnetization transfer ratio values from water frequency.

APTw SI = MTRasym [+3.5 ppm] (%). APTw SI: amide proton transfer weighted percentage, MTRasym [+3.5 ppm] (%): magnetization transfer ratio asymmetry at +3.5 ppm offset frequency.

The ADC was calculated from DWI data (b = 0, 1000 s/mm2) as: ADC=ln(S0/S1)/(b1-b0) where S is the signal intensity at a given b factor, Ssat is the signal intensity obtained with selective saturation, and S0 is the normalization factor acquired at −1540 ppm.

EADC is the indexation result of the signal ratio S/S0, that is e- b·ADC, which focuses more on signal comparison rather than absolute quantification.

Its significance lies in reflecting the signal changes caused by the diffusion behavior of the lesion itself. Moreover, it eliminates the transmission effect of T2W and truly shows the different signal changes of diffusive limitation and diffusive freedom. In cases with limited diffusion, it shows low signal on ADC images but high signal on EADC.

Further, 2 observers with 13 and 5 years of clinical experience in MRI, respectively, and blinded to the patient information measured the resulting parameters individually.

The slice with the tumor area on the axial APT MRI image was selected. In this study, region of interest (ROI) was selected instead of volume of Interest (VOI) because bone and soft tissue tumors are often accompanied by local necrosis and hemorrhage. ROI can more accurately avoid heterogeneous areas of the lesion (such as liquefactive necrotic foci and vascular structures) to reduce measurement errors. Moreover, the VOI segmentation of small-volume lesions using 3.0T MRI is easily affected by magnetic field inhomogeneity, and the average measurement of ROI in multiple slices (the maximum slice of the tumor and the slices above and below it) is more in line with actual clinical operations.

**Figure 1:**
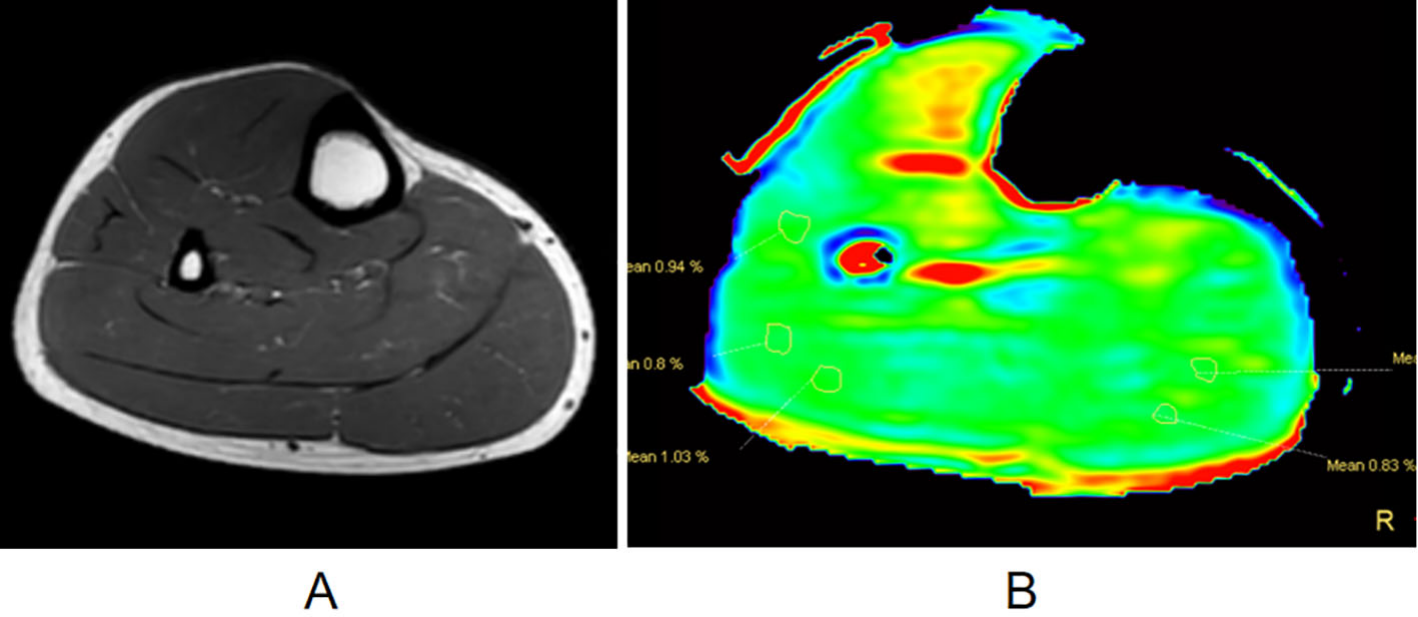
ROI selection method. The tumor was in the tibia. We selected the ROI in the tibia on the APT image. **(A)** Axial T2WI; and **(B)** APT image. APT, Amide proton transfer; ROI, region of interest; T2WI, T2-weighted imaging.

**Figure 2:**
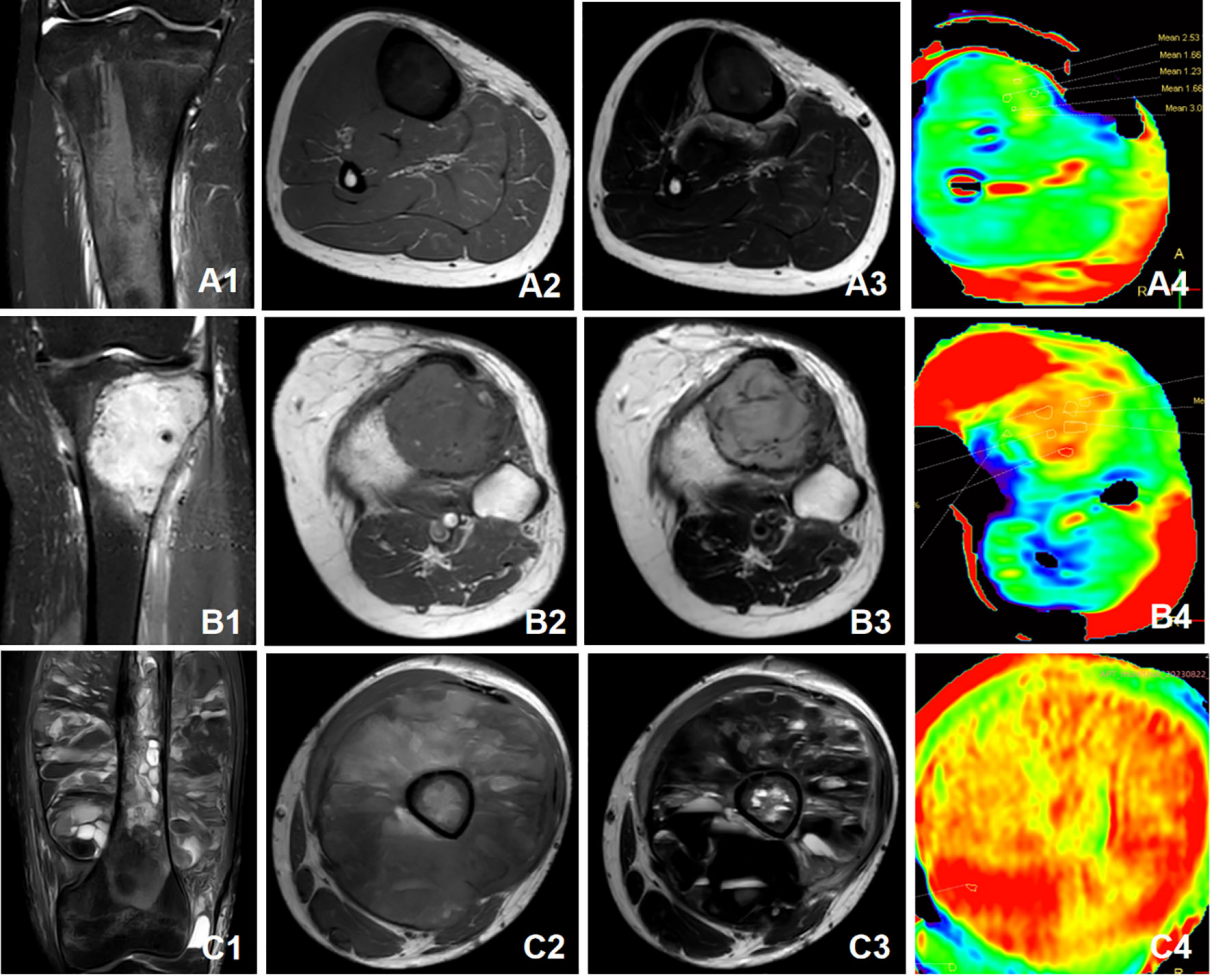
**(A)** Male, 15 years old. The pathological diagnosis was fibrous dysplasia of tibia. A1: Coronal T2-FS sequence showed slightly hyperintensity in the tibia. A2: Axial T1WI showed hypointensity. A3: Axial T2WI showed hypointensity with patchy slightly hyperintensity. A4: APT imaging showed that the mean APT value was 2.02%. **(B)** Female, 63 years old. The pathological diagnosis was Giant cell tumor of bone in the proximal tibia. B1: Coronal T2-FS sequence showed hyperintensity with patchy hypointensity in the lesion. B2: Axial T1WI showed hypointensity. B3: Axial T2WI showed slightly hyperintensity with linear low signal interval. B4: APT imaging showed that the mean APT value was 3.96%. **(C)** Male, 18 years old. The pathological diagnosis was osteosarcoma in the femur. C1: Coronal T2-FS sequence. The lower femur was destroyed, and the surrounding soft tissue mass formed, which manifested as mixed signal. C2: Axial T1WI. C3: Axial T2WI. Many liquid planes were visible on T2WI. C4: APT imaging showed that the mean APT value was 6.32%. APT, Amide proton transfer; FS, Fat suppression; T1WI, T1 weighted image; T2WI, T2 weighted image.

The slice with the tumor area on the axial APT map with fusion onto the fat-suppressed T2WI image was selected. ROI containing the tumor region was manually delineated using a freehand tool to obtain the APT map and then copied to the ADC map and EADC map. The criteria for ROI determination were as follows: the ROI was selected in the maximum, upper and lower slices display of the tumor meanwhile avoiding cystic necrosis and hemorrhage. The average value from the three slices was used for the final value. We have increased these content in the proper location. **Figure 1** illustrates the ROI selection on the APT image. **Figure 2** depicts the MRI features of various patients with benign and malignant tumors.

### Statistical analysis

The pathologic results were obtained by surgical treatment. We compared the statistical differences in APT, ADC, and EADC values between benign and malignant tumors. Statistical analysis was accomplished using the MedCalc 13.0 and SPSS 19.0 software. A 2-way model average measure intraclass correlation coefficient (ICC) was used to test the interobserver agreement, where an ICC >0.75 was considered good. The independent-sample *t* test was used for normally distributed data. The independent-sample Mann–Whitney *U* test, a nonparametric test, was used for the abnormally distributed data.

The correlation among APT, ADC, EADC, and Ki-67 index was assessed using Spearman’s correlation coefficient. The receiver operating characteristic (ROC) curve analysis was performed to evaluate the diagnostic capacity of APT, ADC, and EADC values in distinguishing benign from malignant tumors and Ki-67 expression levels. Meanwhile, Delong tests were conducted to evaluate whether the area under the ROC curve (AUC) of 1 parameter differed from that of the other parameters. The AUC values of <0.7, 0.7-0.9, and >0.9 indicated low, medium, and high diagnostic performance, respectively. A *P* value <.05 was considered statistically significant.

## Results

The ICC was 0.848 in the interobserver agreement, and 95% confidence interval (CI) was 0.607-0.946. The APT, ADC, and EADC values of benign and malignant tumors are depicted in **Table 3**. The APT value was 2.26% ± 1.07% in benign tumors and 4.62% ± 1.43% in malignant tumors. The ADC value was 1.33 ± 0.43 ×10_–3_ mm_2_/s in benign tumors and 1.01 ± 0.55 ×10_–3_ mm_2_/s in malignant tumors. The EADC value was 0.29 ± 0.13 in benign tumors and 0.40 ± 0.17 in malignant tumors. Statistical differences were observed in APT, ADC, and EADC values between benign and malignant tumors (*P* <.001, *P* = .032, and *P* = .019, respectively).

**Table 3:** Comparing the diagnostic efficiency in benign and malignant bone and soft tissue tumors.

Parameter	Benign tumor	Malignant tumor	*P* value
APT (%)	2.26 ± 1.07	4.62 ± 1.43	<.001
ADC (×10_–3_ mm_2_/s)	1.33 ± 0.43	1.01 ± 0.55	.032
EADC	0.29 ± 0.13	0.40 ± 0.17	.019

ADC, Apparent diffusion coefficient; APT, amide proton transfer; EADC, exponential apparent diffusion coefficient.

**Figure 3:**
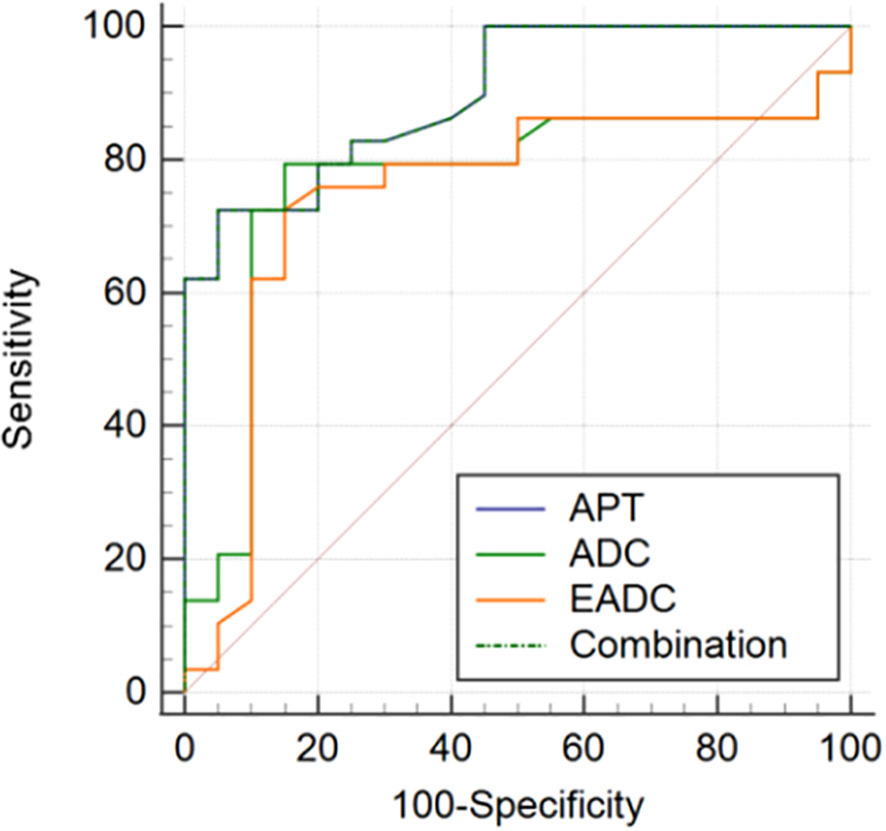
ROC curve of APT, ADC, and EADC values in differentiating benign from malignant bone and soft tissue tumors. APT: AUC was 0.899; sensitivity and specificity were 72.41% and 95%, respectively, and the cutoff value was 3.6% (*P* <.0001). ADC: AUC was 0.765; sensitivity and specificity were 79.31% and 85%, respectively; and the cutoff value was 1.047 ×10_–3_ mm_2_/s (*P* = .0005). EADC: AUC value was 0.745; sensitivity and specificity were 72.41% and 85%, respectively, and the cutoff value was 0.34 (*P* = .019). Combined diagnosis: AUC value was 0.899, and sensitivity and specificity were 72.41% and 95%, respectively (*P* <.001). ADC, Apparent diffusion coefficient; APT, amide proton transfer; AUC, area under the curve; EADC, exponential apparent diffusion coefficient; ROC, receiver operating characteristic.

The sensitivity and specificity of APT, ADC, and EADC in distinguishing between benign and malignant tumors are depicted in **Table 4**, **Figure 3**. In APT analysis for differentiating benign from malignant tumors, the AUC was 0.899, the cutoff value was 3.6%, and the sensitivity and specificity were 72.41% and 95%, respectively (*P* <.0001). In ADC analysis for differentiating benign from malignant tumors, the AUC was 0.765, the cutoff value was 1.047 ×10_–3_ mm_2_/s, and the sensitivity and specificity were 79.31% and 85%, respectively (*P* = .0005). In EADC analysis for differentiating benign from malignant tumors, the AUC was 0.745, the cutoff value was 0.34, and the sensitivity and specificity were 72.41% and 85%, respectively (*P* <.019). The AUC in APT was larger than in ADC and EADC. The sensitivity and specificity were precise in distinguishing between benign and malignant tumors. On combining APT, ADC, and EADC to distinguish between benign and malignant tumors, the AUC was 0.899 and the sensitivity and specificity were 72.41% and 95%, respectively (*P* <.001). However, no statistical differences were observed between the AUCs of APT and ADC and between the AUCs of APT and EADC (*Z* = 1.688, *P* = .0914; *Z* = 1.863, *P* = .0625).

**Table 4:** Sensitivity and specificity of APT, ADC, and EADC in distinguishing between benign and malignant tumors.

Parameter	Sensitivity (%)	Specificity (%)	Cutoff value	AUC	*P* value
APT	72.41	95	3.6	0.899	<.0001
ADC	79.31	85	1.047	0.765	.0005
EADC	72.41	85	0.34	0.745	.019
Combined diagnosis	72.41	95	/	0.899	<.001

ADC, Apparent diffusion coefficient; APT, amide proton transfer; AUC, area under the curve; EADC, exponential apparent diffusion coefficient.

**Figure 4:**
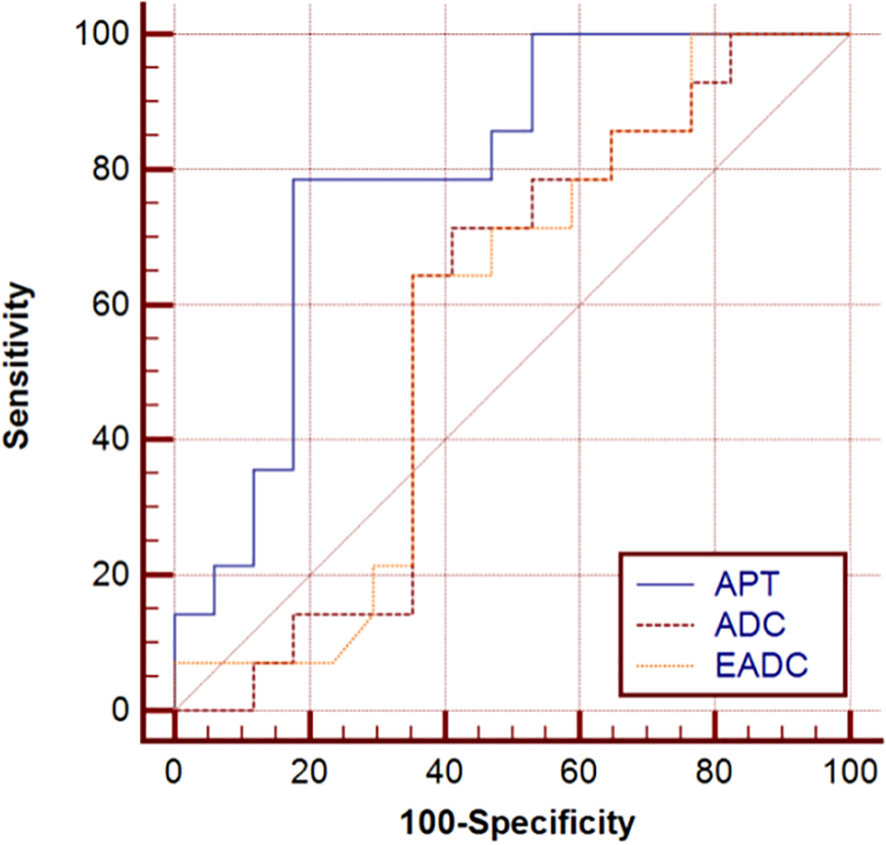
Diagnostic efficiency of APT, ADC, and EADC values in assessing Ki-67 expression was evaluated using the ROC curve. APT: AUC was 0.794, and sensitivity and specificity were 100% and 57.14%, respectively (*P* <.0001). ADC: AUC was 0.576, and sensitivity and specificity were 70.59% and 85.71%, respectively (*P* <.0001). EADC: AUC value was 0.578, and sensitivity and specificity were 70.59% and 85.71%, respectively (*P* <.0001). ADC, Apparent diffusion coefficient; APT, amide proton transfer; AUC, area under the curve; EADC, exponential apparent diffusion coefficient; ROC, receiver operating characteristic.

Further, the Ki-67 expression was examined through immunohistochemistry in 31 patients. Among the 31 patients 15 cases were benign tumors and 16 cases were malignant tumors. There was no significant difference in the distribution of age and tumor type between these 31 patients and the total sample (49 cases) (P = .341, P = .112, respectively), which initially rules out selection bias. The relationship among APT, ADC, EADC, and Ki-67 index are presented in **Table 5**. APT exhibited a positive correlation with Ki-67 [Spearman correlation coefficient: *r* = 0.546, 95% confidence interval (CI): 0.217-0.762, *P* = .002]; ADC exhibited no correlation with Ki-67 (*r* = −0.260, 95% CI: −0.503 to 0.030, *P* = .157); and EADC exhibited no correlation with Ki-67 (*r* = 0.269, 95% CI: −0.097 to 0.593, *P* = .144). **Figure 4** demonstrates the diagnostic efficiency of APT, ADC, EADC, and the Ki-67 index. The AUC of APT, ADC, and EADC with Ki-67 was 0.794, 0.576, and 0.578, respectively, and the 95% CI was 0.611-0.917, 0.386-0.750, and 0.388-0.752, respectively.

**Table 5:** Relationship between APT, ADC, and EADC values and Ki-67 expression in 31 patients with benign and malignant tumors.

Parameter	Benign tumor	Malignant tumor	*P* value	*P* value (with Ki-67)
APT %	2.29 ± 1.38	4.73 ± 1.45	<.001	.002
ADC (×10–^3^ mm^2^ /s)	1.10 ± 0.60	0.99 ± 0.67	.630	.157
EADC	0.39 ± 0.20	0.43 ± 0.21	.607	.144
Ki-67 %	19.00 ± 20.96	45.94 ± 28.36	.005	/

ADC, Apparent diffusion coefficient; APT, amide proton transfer; EADC, exponential apparent diffusion coefficient.

## Discussion

In this study, the APT and EADC values were higher and the ADC value was lower in malignant bone and soft tissue tumors than in benign tumors. Statistical differences were observed in APT, ADC, and EADC values between benign and malignant tumors. In differentiating between benign and malignant tumors, when the APT value was 3.6%, the sensitivity and specificity were 72.41% and 95%, respectively; when the ADC value was 1.047 ×10–^3^ mm^2^/s, the sensitivity and specificity were 79.31% and 85%, respectively; when the EADC value was 0.34, the sensitivity and specificity were 72.41% and 85%, respectively. Combining the APT method with DWI indicated higher sensitivity and specificity for qualitatively diagnosing benign and malignant bone and soft tissue tumors. The diagnostic efficiency of the APT value was higher than that of ADC and EADC in assessing the Ki-67 expression.

Tumor characteristics are assessed using the TNM stage, which affects tumor size, the number of lymph nodes, and the presence or absence of metastasis. These factors are associated with the risk of tumor aggressiveness and recurrence, but fail to predict tumor aggressiveness at an early stage. However, evaluating immunohistochemical protein markers such as tumor protein p53 and Ki-67 index can help predict the tumor biologic behavior and recurrence at an early stage after surgery (22).

Conventional MRI mainly provides the morphologic information of tumors; however, sometimes, it cannot differentiate benign from malignant bone and soft tissue tumors. DWI helps differentiate benign from malignant tumors by providing insight into the cellularity with ADC and EADC images (1). Jhaveri suggested that DWI provides complementary biophysical information on tumor microstructure and can be used for further evaluating rectal cancer grading and predicting response to chemoradiotherapy (23).

APT is geared toward detecting the exchangeable amide protons in the backbone of mobile proteins (15). Studies indicate a strong correlation between APT signal intensity and increased cell density, proliferation, and concentrations of intracellular protein in gliosarcoma and breast cancer (12, 14). A high signal intensity in APT image often indicates active metabolism of tumors and higher protein concentration.

A study revealed that the proliferation rate of malignant tumors increases rapidly, the content of protein and polypeptide in tumor cells increases, and the chemical exchange saturation transfer effect is enhanced (24). These results were consistent with increasing protein concentrations in malignant tumors, as revealed by MRI-guided proteomics (25, 26) and *in vivo* MR spectroscopy (25) in several studies. In contrast, the proliferation rate of benign tumor cells is slow, the content of protein and polypeptide is relatively less than that of malignant tumor cells, and the effect of chemical exchange saturation metastasis is weaker than that of malignant tumors. Therefore, the APT value in the benign tumor was lower than that in the malignant tumor in this study. The mobile protein in the blood generates strong endogenous APT contrast as inherent problems (27). Another possible contribution to increased protein signal in malignant tumors is angiogenesis, as the blood contains high concentrations of hemoglobin and albumin (28).

The findings of this study demonstrated that the APT value was lower in benign bone and soft tissue tumors than in malignant tumors. Based on the ROC curve, when the APT value was 1.455%, the sensitivity and specificity of the diagnosis in the benign tumors were 68.42% and 100%, respectively. When the APT value was 3.6%, the sensitivity and specificity of the diagnosis in the malignant tumors were 86.36% and 100%, respectively. Zhou Liu et al. (29) demonstrated that the APT signal was considerably higher in malignant lesions than in benign lesions in breast cancer. Osamu Togao suggested that APT imaging can predict the histopathologic grades of adult diffuse gliomas. The mean APT signal intensity (SI) values were 2.1% ± 0.4% in grade II gliomas, 3.2% ± 0.9% in grade III gliomas, and 4.1% ± 1.0% in grade IV gliomas. Significant differences were observed in APT intensity between grades II and III (*P* <.05) and grades III and IV (*P* <.05), as well as between grades II and IV (*P* <.001) (27). Our study also revealed that the APT imaging differentiated between benign and malignant bone and soft tissue tumors. A significant difference was observed in the benign and malignant bone and soft tissue tumors. The APT value was lower in benign tumors than in malignant tumors.

In any event, an intracellular increase in pH would be synergistic for tumor detection. Acidic extracellular pH caused by lactate removal from the cell (30) suggests that the proteins and peptides of interest for the APT contrast are primarily located inside the tumor cell because the amide exchange rate reduces substantially at lower pH. Tumor tissues are prone to hypoxic–ischemic necrosis due to the rapid growth of malignant tumors, resulting in the formation of a local acidic environment (31) and a decrease in the pH value. The exchange rate between amide protons and water particles is reduced. However, proteins and peptides are released to the surrounding areas during tissue necrosis (32), increasing endogenous protein content and promoting the exchange rate of amide protons and water neutrons. The effect of this process on APT value may be higher than that of the decrease in pH value, resulting in a higher APT value of malignant tumors.

Fatty tissue or fluids, such as cysts, blood vessels, or hemorrhage, may exhibit hyperintensity on APT imaging due to their high protein content. When reviewing APT images, referring to routine structural MR images to identify “hyperintensity artifacts,” such as liquefactive necrosis, cysts, hemorrhages, and vessels, is necessary for an accurate interpretation. Therefore, in our study, we avoided liquefactive necrosis, cysts, hemorrhages, and vessels when determining the ROI position for the tumor.

APT images exhibit high signal intensity in areas with high cell density, elevated cytoplasmic protein levels, and increased intercellular pH value (33, 34). Meanwhile, malignant tumors have high Ki-67 expression. Therefore, the higher the Ki-67 index, the higher the density of tumor cells and the higher the APT signal intensity. Our study observed a positive correlation between the APT value and the Ki-67 expression. Li et al. (21) demonstrated that the APT value was related to p53 and Ki-67 expression levels in rectal adenocarcinoma. APT imaging may serve as a noninvasive biomarker for assessing genetic prognostic factors of rectal adenocarcinoma. Zhou Liu et al. (29) suggested that the APT signal significantly correlated with the Ki-67 index in breast cancer. APT imaging revealed its potential in differentiating breast lesion malignancy and association with prognosis-related tumor grade, T stage, and proliferative activity. We obtained similar results in the correlation between the APT value and the Ki-67 index.

In evaluating the relationship between ADC value and Ki-67 expression, we observed that the higher the Ki-67 index, the lower the ADC value in all tumors. ADC measures the extent of water molecule diffusion in the tissue. The decreased ADC value was due to the increase in nuclear size, cytoplasmic protein, and cytoplasmic viscosity (35). Tumors with high Ki-67 expression have high proliferative activity and increased cell density, limiting water diffusion. This explains the decrease in ADC value. A previous meta-analysis demonstrated a statistically significant negative association between ADC and Ki-67 across various tumors (36). Several studies reporting on the relationship between ADC and colorectal cancer Ki-67 only demonstrated a weak-to-moderate correlation. The association between ADC and Ki-67 has also been reported in soft tissue tumors. For example, Fabian Schmitz et al. (37) found mean ADC was lower in high proliferative than low proliferative soft tissue sarcomas, as low ADC values represent hypercellularity. However, the difference was small and insignificant. Some other studies showed a negative correlation with ADC for Ki-67 in the murine model of rhabdomyosarcoma and human soft tissue sarcoma patients (38–42), which were consistent with the trend of the results in this study (r=-0.260, P = .157), suggesting the limitations of ADC in evaluating the proliferative activity of soft tissue tumors. ADC was susceptible to microcirculation perfusion, motor aberration, and sensitivity differences (35). Although several studies have demonstrated that ADC effectively evaluates the tumor proliferation factor Ki-67, its use as an alternative marker to predict tumor proliferation is limited (36). The radiologic features and histogram parameters based on multiparameter MRI are also useful for evaluating Ki-67, but are not reliable enough (43–45). In our study, no statistical differences were observed in the correlation among ADC, EADC, and Ki-67 expression. This may be attributed to the small number of cases, which affected the results.

In our study, the ROC curve demonstrated that the APT value had the highest diagnostic ability in predicting the Ki-67 expression compared with the ADC and EADC values. We demonstrated that the APT value could provide improved diagnostic capability compared with the ADC and EADC values in assessing the Ki-67 expression. Li et al. (21) demonstrated APT and DWI in assessing Ki-67 expression in rectal adenocarcinoma. Their results from ROC analysis revealed that APT SI had the diagnostic ability in predicting various Ki-67 expression levels, whereas ADC demonstrated only a weak predictive ability. Although this study was novel in using APT, ADC, and EADC with Ki-67 expression to diagnose bone and soft tissue tumors, the results were consistent with those of the aforementioned studies.

The impact of histological heterogeneity on APT is mainly reflected in the fact that “the APT value of well-differentiated tumors (such as low-grade liposarcoma) is close to that of benign tumors”. However, in this study, “pathological grade stratified analysis” showed that the APT values of well differentiated malignant tumors were still significantly higher than those of benign tumors (P <.01), indicating that heterogeneity did not obscure the difference between benign and malignant tumors.

## Limitations

This study had several limitations. First, our study had a relatively small sample size. This study is a single-center study. In the future, the sample size should be expanded through multi-center studies for further analysis to validate the results. Second, conventional assessments, such as T1WI and T2WI imaging characteristics, have limitations in differentiating benign from malignant tumors, so they were not within the scope of our study. Third, we preliminarily analyzed the APT signal intensity value in benign and malignant bone and soft tissue tumors, and whether tumors from various tissue sources have various APT characteristics needs to analyze. In future studies, the sample size can be expanded, and stratified analysis can be carried out according to the tissue origin of the tumor (bone/soft tissue) to further improve the pertinence of the results. The positive correlation between APT and Ki-67 in this study (r=0.546, P = .002) should be interpreted with caution. Since only 31 patients were included, the sample size of Ki-67 detection should be expanded in the future to verify the stability of this association. Finally, we analyzed the correlation among APT, ADC, and EADC values and the pathologic proliferation index Ki-67 expression, but the patients exhibited no postoperative MRI follow-up using APT and DWI scanning. In future studies, the MRI relative indexes need to be followed up at various time intervals, especially in patients with malignant tumors, to verify the correlation among APT, ADC, and EADC values and the survival cycle of these patients. Therefore, we suggest using preoperative APT imaging to help predict the survival cycle of patients with malignant tumors.

## Conclusions

The MR APT sequence has high qualitative diagnostic efficiency in differentiating benign and malignant bone and soft tissue tumors (when the cutoff value is 3.6%, the sensitivity is 72.41% and the specificity is 95%); the combination of APT, ADC and EADC can improve the diagnostics specificity; the APT value is positively correlated with Ki-67 expression (r = 0.546, P = .002), and can be used as a non-invasive imaging marker to evaluate the proliferative activity of bone and soft tissue tumors. These results need to be further verified by large-sample and multi-center studies.

There were no funding in this study.

## Data Availability

The original contributions presented in the study are included in the article/supplementary material. Further inquiries can be directed to the corresponding author.
